# Impact of fetal presentation on neurodevelopmental outcome in a trial of preterm vaginal delivery: a nationwide, population-based record linkage study

**DOI:** 10.1007/s00404-021-06146-z

**Published:** 2021-10-31

**Authors:** Anna Toijonen, Seppo Heinonen, Mika Gissler, Laura Seikku, Georg Macharey

**Affiliations:** 1 Department of Obstetrics and Gynecology, University of Helsinki, Riihiuunintie 12 c, 02620 Espoo, Finland; 2 Department of Obstetrics and Gynecology, University Hospital (HUS), University of Helsinki, Haartmaninkatu 2, 00290 Helsinki, Finland; 3 National Institute for Health and Welfare (THL), Helsinki, Finland; 4 Department of Obstetrics and Gynecology, University Hospital (HUS), University of Helsinki, Haartmaninkatu 2, 00290 Helsinki, Finland

**Keywords:** Preterm delivery, Preterm labor, Breech presentation, Vaginal labor, Adverse outcome

## Abstract

**Purpose:**

To assess the risk of adverse neurodevelopmental outcomes at the age of four after an attempted vaginal delivery according to the fetal presentation in birth.

**Methods:**

This retrospective record linkage study evaluated the risks of cerebral palsy, epilepsy, intellectual disability, autism spectrum disorder, attention-deficit/hyperactivity disorder, and speech, visual, and auditory disabilities among preterm children born after an attempted vaginal breech delivery. The control group comprised children born in a cephalic presentation at the same gestational age. This study included 23 803 singleton deliveries at gestational weeks 24 + 0–36 + 6 between 2004 and 2014.

**Results:**

From 1629 women that underwent a trial of vaginal breech delivery, 1122 (66.3%) were converted to emergency cesarean sections. At extremely preterm and very preterm gestations (weeks 24 + 0—31 + 6), no association between a trial of vaginal breech delivery and neurodevelopmental delay occurred. At gestational weeks 32 + 0—36 + 6, the risks of visual disability (aOR 1.67, CI 1.07—2.60) and autism spectrum disorders (aOR 2.28, CI 1.14—4.56) were increased after an attempted vaginal breech delivery as compared to vaginal cephalic delivery.

**Conclusion:**

A trial of vaginal breech delivery at extremely preterm and very preterm gestations appears not to increase the risk of adverse neurodevelopmental outcomes at the age of four. In moderate to late preterm births, a trial of vaginal breech delivery was associated with an increased risk of visual impairment and autism spectrum disorders compared to children born in cephalic presentation. A trial of vaginal preterm breech delivery requires distinctive consideration and careful patient selection.

**Supplementary Information:**

The online version contains supplementary material available at 10.1007/s00404-021-06146-z.

## Introduction

Preterm birth may significantly compromise the child's long-term neurodevelopment [1]. In Finland, preterm birth occurs in approximately five percent of singleton pregnancies [2], and depending on the gestational age, from 2.5 to 23.5% of these fetuses are in breech presentation [3].

A trial of preterm vaginal delivery in cephalic presentation is generally accepted management [4]. Nonetheless, the optimal delivery mode in preterm breech presentation is unknown, as the literature shows contradictory risks concerning neonatal mortality and morbidity [5–10]. In term pregnancies, vaginal breech delivery is widely accepted, with exceptions regarding pregnancies with certain risk factors such as oligohydramnios, fetal growth restriction, or otherwise compromised fetus [11–13]. Vaginal breech delivery at term is not associated with abnormal childhood neurodevelopment or with increased risk of epilepsy as compared to delivery by cesarean section [14, 15]. However, several risk factors have been associated with a preterm breech presentation [3]. The risk factors for breech presentation vary according to gestational age [3], and many of these factors are linked to neonatal adverse outcomes, especially in vaginal delivery [16]. Several studies have demonstrated an increased risk of delayed intellectual or neuromotor development among preterm children born vaginally in breech presentation [5–7]. However, the findings are not solid as planned cesarean section has not improved the outcomes of preterm breech deliveries in all studies [9]. Due to the difficulties in arranging randomized controlled trials and the numerous confounding factors affecting observational studies, further studies are needed to provide evidence on the optimal management of preterm breech deliveries.

Our study investigates the impact of fetal presentation on neurodevelopmental outcomes at the age of four.

## Methods

We conducted a population-based record linkage study on all children born between 24 + 0 and 36 + 6 gestational weeks from 2004 to 2014 in Finland. The Finnish Institute for Health and Welfare offered and authorized the data, as the Finnish national data protection law requires (reference number THL/652/5.05.00/2017). The data included the Medical Birth Register and the Hospital Discharge Register with the information on all surgical procedures and diagnoses (International Statistical Classification of Diseases and Related Health Problems 10^th^ Revision, ICD-10) in inpatient care (all hospitals) and outpatient care (public hospitals). All the maternity hospitals are obligated to serve the data to the national registers.

We excluded multiple gestations, term deliveries, and deliveries without the information on gestational age or fetal presentation. Only deliveries with a fetus either in breech or cephalic presentation were included. We limited the study to preterm deliveries of ≥ 24 + 0 gestational weeks and excluded the pregnancies with placental abruption (ICD-10 O71.0, O71.1) or major congenital anomalies (as defined in the Register of Congenital Malformations), as such conditions and earlier gestational ages with lower viability of the infant might affect the results. As we aimed to evaluate the long-term effects of an attempted vaginal delivery according to the fetal presentation, we excluded the planned cesarean sections from our study.

We stratified the study population into three groups according to the World Health Organization's sub-categories of preterm birth: extremely preterm birth (gestational age 24 + 0–27 + 6), very preterm birth (gestational age 28 + 0–31 + 6), and moderate to late preterm birth (gestational age 32 + 0–36 + 6). Each sub-category of preterm delivery was separately adjusted. The control groups comprised children born preterm in a cephalic presentation at comparable gestational ages. Odds ratios (OR) with 95% confidence intervals (Cl) estimated the relative risk of neurodevelopmental disability at the age of four. The primary outcomes were neurodevelopmental disabilities: cerebral palsy (CP), epilepsy, intellectual disability (ID), autism spectrum disorder (ASD), attention-deficit/hyperactivity disorder (ADHD), speech, visual (ICD-10 H00-59), and auditory problems (ICD-10 H60-95). Late neonatal deaths at 28–364 days from birth were evaluated. As we aimed to examine the long-term effects of an attempted vaginal breech delivery, we did not include neonatal deaths or other short-term outcomes in this research.

The analysis included the following maternal variables: age, smoking, parity, pre-pregnancy body mass index (BMI), history of cesarean section, hypo- and hyperthyroidism (ICD-10 E03, E05), pre-gestational diabetes (ICD-10 O24.0, O24.1), gestational diabetes (ICD-10 O24.4), preeclampsia, and pregnancy-induced hypertension (ICD-10 O13, O14). The following obstetric and fetal confounders were also acknowledged: oligohydramnios (ICD-10 O41.0), fetal sex, birthweight below third and 10th percentile of standard deviation, preterm premature rupture of membranes (PPROM) (ICD-10 O42), induction of labor, epidural analgesia (apart from epidural anesthesia in emergency cesarean section), and emergency cesarean section after a trial of vaginal breech delivery.

The statistical calculations were performed by SAS 9.4. We used a multivariate logistic regression model and chi-Squared test or Fisher's exact test when appropriate to adjust for confounders. *p* values of ≤ 0.05 were statistically significant.

## Results

**Fig. 1:**
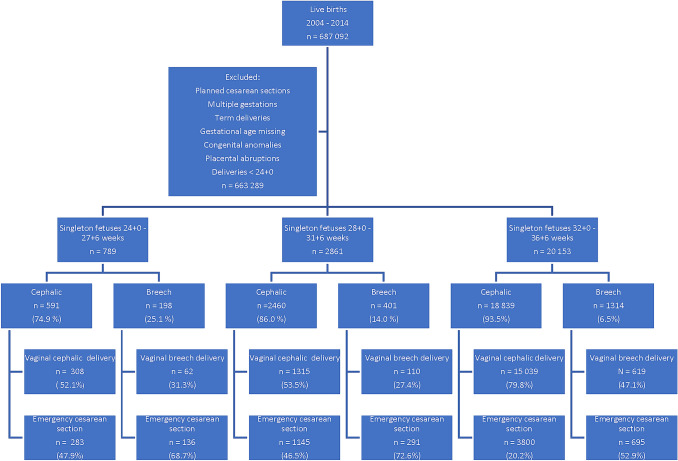
A flowchart of the study population, preterm deliveries (vaginal and emergency cesarean sections) 2004–2014 in Finland

We analyzed 23 803 preterm singleton deliveries. After exclusions, the study included 19 430 preterm fetuses, and 1629 (8.4%) of these were in breech presentation at the time of birth. The percentage of the breech presentation decreased from 25.1 to 6.5% by advancing gestational age. (Fig. 1).

The group of extremely preterm fetuses (gestational age 24 + 0–27 + 6) included 789 live births. An emergency cesarean section was performed in 68.7% (*n* = 136) pregnancies with a fetus in breech presentation and in 47.9% (*n* = 283) in cephalic presentation (OR 2.39; 95% CI 1.70–3.36, p-value < 0.001). The breech deliveries in extremely preterm were less likely induced than cephalic deliveries (OR 0.23; 95% CI 0.08–0.67, *p* value 0.003). Otherwise, the groups in extremely preterm did not differ significantly in maternal, fetal, or obstetric characteristics. Additional data on characteristics are given in Online Resource 1. At the age of four, the children with a trial of vaginal delivery in extremely preterm did not differ in neurodevelopment between the breech and cephalic presentation groups. The most common disabilities of these children were speech development delay (breech 9.1%, cephalic 13.4%) and cerebral palsy (breech 5.1%, cephalic 4.4%). Neonatal mortality within 28 to 364 days was 3.5% among the breech group and 2.7% in the cephalic group. The differences in mortality were statistically insignificant. (Table 1).

**Table 1:** At the age of four, children's neurodevelopmental outcomes after an attempted vaginal delivery in 24 + 0 to 36 + 6 gestational weeks

	24 + 0–27 + 6			28 + 0–31 + 6			32 + 0–36 + 6		
	Breech *n* = 198 (%)	Cephalic *n* = 591 (%)	aOR (95% CI)	Breech *n* = 401 (%)	Cephalic *n* = 2460 (%)	aOR (95% CI)	Breech *n* = 1314 (%)	Cephalic *n* = 18 839 (%)	aOR (95% CI)
CP	10 (5.1%)	26 (4.4%)	1.31 (0.61–2.82)	12 (3.0%)	65 (2.6%)	1.36 (0.71–2.57)	2 (0.2%)	81 (0.4%)	0.25 (0.06–1.11)
Epilepsy	2 (1.0%)	12 (2.0%)	0.42 (0.09–1.92)	8 (2.0%)	26 (1.1%)	1.70 (0.74–3.91)	6 (0.5%)	119 (0.6%)	0.77 (0.33–1.78)
ID	2 (1.0%)	9 (1.5%)	0.61 (0.13–2.91)	4 (1.0%)	26 (1.1%)	1.12 (0.38–3.31)	2 (0.2%)	64 (0.3%)	0.43 (0.10–1.79)
ASD	2 (1.0%)	11 (1.9%)	0.50 (0.11–2.31)	4 (1.0%)	19 (0.8%)	1.51 (0.49–4.63)	10 (0.8%)	70 (0.4%)	2.28 (1.14–4.56)
Speech	18 (9.1%)	79 (13.4%)	0.65 (0.38–1.13)	17 (4.2%)	112 (4.6%)	0.90 (0.53–1.53)	31 (2.4%)	473 (2.5%)	0.91 (0.63–1.33)
Visual	6 (3.0%)	26 (4.4%)	0.70 (0.28–1.76)	6 (1.5%)	55 (2.2%)	0.66 (0.28–1.57)	24 (1.8%)	206 (1.1%)	1.67 (1.07–2.60)
Auditory	0 (0.0%)	4 (0.7%)		2 (0.5%)	13 (0.5%)	0.94 (0.21–4.31)	3 (0.2%)	55 (0.3%)	0.56 (0.17–1.84)
ADHD	1 (0.5%)	9 (1.5%)	0.25 (0.03–1.98)	1 (0.2%)	18 (0.7%)	0.30 (0.04–2.34)	7 (0.5%)	53 (0.3%)	1.82 (0.80–4.17)
Child death 28–364 days	0 (0.0%)	14 (0.6%)					3 (0.2%)	25 (0.1%)	1.49 (0.43–5.14)

24 + 0–27 + 6 adjusted for emergency cesarean section and induction of labor

28 + 0–31 + 2 adjusted for maternal age < 25, parity ≥ 3, PPROM, induction of labor, epidural analgesia, and emergency cesarean section

32 + 0–36 + 6 adjusted for maternal age < 25, smoking, primiparity, parity ≥ 3, maternal BMI < 20, maternal hyperthyroidism, pre-gestational diabetes, gestational diabetes, oligohydramnios, female sex, birthweight < 10%, PPROM, induction of labor, epidural analgesia, and emergency cesarean section

*CP* cerebral palsy; *ID* intellectual disability; *ASD* autism spectrum disorder; *ADHD* attention-deficit/hyperactivity disorder

The group of very preterm birth (gestational age 28 + 0–31 + 6) included 2861 deliveries. Of all very preterm fetuses in breech, 72.6% (*n* = 291) were born by emergency cesarean section, and of the cephalic fetuses, 46.5% (*n* = 1145) (OR 3.03, 95% CI 2.41–3.84, *p* value < 0.001). In this very preterm group, the mothers with a fetus in breech presentation were more likely to be above 25 years old at the time of delivery and non-primipara than the mothers in the cephalic group. Multiparity (≥ 3 deliveries) and PPROM were more common in the breech group than in the cephalic presentation group. Furthermore, the breech deliveries had fewer inductions and epidural analgesia compared to cephalic deliveries (Online Resource 2). The children born after a trial of vaginal breech delivery in this very preterm group had no increased risk of neurodevelopmental disabilities than the cephalic deliveries. Speech development problems (breech 4.2%, cephalic 4.6%) and cerebral palsy (breech 3.0%, cephalic 2.6%) appeared to be the most frequent problems among children in the study group. Among the breech children, no later deaths in 28–364 days occurred, whereas, among the children born in cephalic presentation, fourteen such deaths (0.6%) occurred. The difference in mortality was not significant. (Table 1).

Our study enclosed 20 153 moderate to late preterm deliveries (gestational age 32 + 0–36 + 6). An emergency cesarean section was the final mode of delivery in half of the breech pregnancies (52.9%) and one-fifth of the cephalic ones (20.2%) (OR 4.44, 95% CI 3.96–4.98, *p* value < 0.001). Primiparity, maternal hyperthyroidism, oligohydramnios, PPROM, fetal female sex, and birthweight below 10% were more common in the breech presentation group than in the cephalic group in these moderate to late preterm deliveries. Accordingly, the mothers in the breech presentation group were more likely at least 25 years old, non-smokers, obese (BMI ≥ 20), and non-diabetes (pre-gestational or gestational diabetes) than the mothers in the cephalic group. The breech deliveries were less likely induced or needed epidural analgesia compared to cephalic deliveries (Online Resource 3). Breech presentation in a trial of vaginal delivery doubled the risk of autism spectrum disorders (aOR 2.28, CI 1.14–4.56) and the visual defects were significantly increased (aOR 1.67, CI 1.07–2.60). Among the breech children born at moderate to late preterm gestations, three deaths (0.2%) occurred in 28 to 364 days. There were twenty-five (0.1%) late neonatal deaths in the cephalic group. (Table 1).

## Discussion

We demonstrated that a trial of vaginal breech delivery at extremely preterm and very preterm gestations do not increase the risk of adverse neurodevelopmental outcome at the age of four. However, among moderate to late preterm births, a trial of vaginal breech delivery associates with an increased risk of visual impairment and autism spectrum disorders compared to children born in cephalic presentation.

In term delivery, studies have not connected vaginal breech delivery to a higher CP risk than in cephalic deliveries [20, 21]. However, prematurity exposes the child to substantial morbidity and mortality [1]. The literature presents controversial results on short-term outcomes of vaginal preterm breech delivery. Schmidt and colleagues favored cesarean section for breech deliveries of less than 32 weeks of gestation to minimize neonatal morbidity [8]. In a Swedish study, a vaginal breech delivery at extremely preterm gestation increased the risk of neurodevelopmental delay [5]. Wood and colleagues reported similar results by demonstrating an association between extremely preterm vaginal breech delivery and cerebral palsy, as well as with severe neuromotor disability [6]. Furthermore, an extremely preterm vaginal breech delivery is connected to delayed neuropsychological development [7]. On the contrary, we found no excess/additional risk of neuromotor developmental delay after a trial of vaginal breech delivery at extremely or very preterm gestations.

Our findings concerning visual impairments after a trial of vaginal breech delivery among moderate to late preterm births are in line with a previous study indicating an elevated risk of retinopathy of prematurity, chronic lung disease, and mortality in vaginal preterm breech delivery [17]. The most important risk factors related to visual problems such as retinopathy of prematurity are chronic intrauterine stress and asphyxia [18]. The increased risk of visual impairments among children born in breech may be explained by intrapartum complications such as difficulties at delivering the after-coming head. Furthermore, in moderate to late preterm pregnancies, breech presentation is associated with several obstetric problems such as oligohydramnios, PPROM, and growth restriction [3]. Various underlying complications may influence the risks of visual impairment and autism spectrum disorders detected in this study group. Moreover, visual symptoms are common among patients with autism spectrum disorders owing to the underlying central nervous system disability [19].

Our study showed no excess mortality among preterm children after a trial of vaginal breech delivery. This finding contradicts several other studies recommending cesarean section to reduce short-term mortality of preterm breech neonates [5, 17, 22, 23, 24]. Some studies align with our findings, indicating no excessive neonatal mortality after a trial of vaginal preterm breech delivery [9, 10].

In our study, epilepsy, intellectual disability, ADHD, speech, or auditory difficulties at the age of four were not increased among preterm children after a trial of vaginal breech delivery. In line with our findings, a recent study on vaginal preterm breech delivery found no association with neurodevelopmental impairment at the age of 2 years [9]. On the contrary, Källén and colleagues (2015) revealed a higher risk of intellectual developmental delay at 2.5 years in children born extremely preterm vaginally in breech presentation [5]. In our study, the breech delivery was more often converted to emergency cesarean sections than cephalic deliveries. This might have had a protective effect on the neurodevelopment as a cesarean section is readily performed if breech delivery is not proceeding expectedly. However, because the follow-up was until the age of four only, there is a possibility that the follow-up time was too short to detect minor differences in epilepsy, intellectual disability, ADHD, speech, or auditory difficulties detected later in life.

### Limitations and strengths

Our study elucidated the long-term effects of vaginal preterm breech delivery during ten years with more than 20 000 breech deliveries. Our study's major strengths are the long follow-up time and highly equal management of the deliveries and the neonatal and pediatric health care at the Finnish public hospitals.

Our study shows that breech children born after a trial of vaginal delivery at gestational ages between 32 + 0 and 36 + 6 weeks had an increased risk of visual impairment and autism spectrum disorders compared to the reference group comprising children born in cephalic presentation. However, it remains unclear whether the attempted vaginal breech delivery or the breech presentation itself increases the risk. The limitations of the study are related to the use of the registered data: We could not distinguish the severity of the visual disability, and we could not examine the duration of the trial of vaginal breech delivery or the delivery stage before converting into emergency cesarean section, since the data were not collected in the Medical Birth Register before 2017. Further caution in the interpretation of the analyses must be considered as multiple comparisons were made in small subgroups. There is a possibility that the study was underpowered to detect all the differences within the selected populations. However, this approach was justifiable because of the variations in the risk profiles between these subgroups. Some outcomes and analyzed factors were present only in a small population and thus broadened the confidence intervals.

## Conclusion

Our study revealed key findings on long-term outcomes of preterm vaginal deliveries and strengthened the concept of vaginal breech births' safety. The results also show that the data from term deliveries cannot be directly used on preterm deliveries.

The risks of visual impairment and autism spectrum disorders are increased among breech children after a trial of vaginal delivery at the gestational age between 32 + 0 and 36 + 6 weeks compared to the reference group with cephalic delivery. No association between a trial of vaginal breech delivery at gestational weeks 24 + 0—31 + 6 and neurodevelopmental delay occurred. A trial of a vaginal breech delivery can be considered for individually selected preterm deliveries with the mother's consent.

## Supplementary Information

Below is the link to the electronic supplementary material.

Supplementary file1 (DOCX 15 KB)

Supplementary file2 (DOCX 15 KB)

Supplementary file3 (DOCX 15 KB)

## Data Availability

The Finnish register data have been given for this specific study, and the data cannot be shared without authorization from the register keepers. More information on the authorization application to researchers who meet the criteria for access to confidential data can be found at Findata, the Health and Social Data Permit Authority: https://www.findata.fi/en/

## Abbreviations

ICD-10: International Statistical Classification of Diseases and Related Health Problems 10th Revision;
OR: Crude odds ratio;
Cl: Confidence interval;
CP: Cerebral palsy;
ID: Intellectual disability;
ASD: Autism spectrum disorder;
ADHD: Attention-deficit/hyperactivity disorder;
NIUT: Neonatal intensive care unit;
BMI: Body mass index;
PPROM: Preterm premature rupture of membranes;
aOR: Adjusted odds ratio

## Code availability

SAS custom code cannot be shared publicly, but it is available on request from Mika Gissler (mika.gissler@thl.fi).
