# Supplementary material for: Impact of fetal presentation on neurodevelopmental outcome in a trial of preterm vaginal delivery: a nationwide, population-based record linkage study
Source: Arch Gynecol Obstet. 2021 Oct 31;306(1):29–35. doi: 10.1007/s00404-021-06146-z (PMC9300511; doi:10.1007/s00404-021-06146-z)
Supplement: Supplementary file 1 — Supplementary file1 (DOCX 15 KB) [file 404_2021_6146_MOESM1_ESM.docx]

**Archives of Gynecology and Obstetrics**

**Impact of fetal presentation on neurodevelopmental outcome in a trial of preterm vaginal delivery: a nationwide, population-based record linkage study**

Toijonen A (University of Helsinki, [anna.toijonen@helsinki.fi](mailto:anna.toijonen@helsinki.fi)), Heinonen S, Gissler M, Seikku L, Macharey G

**Online Resource 1.**

Characteristics of pregnancies that underwent an attempted vaginal delivery in 24+0 to 27+6 gestational weeks 2004-2014 in Finland.

|  | **Breech**  **24+0 - 27+6** | | **Cephalic**  **24+0 - 27+6** | |  |  |  |
| --- | --- | --- | --- | --- | --- | --- | --- |
|  | **n = 198** | **%** | **n = 591** | **%** | **p-value** | **Odds ratio** | **95% confidence interval** |
| Maternal age < 25 years | 8 | 4.0% | 18 | 3.0% | 0.497 | 1.34 | 0.57 - 3.13 |
| Maternal age ≥ 35 years | 48 | 24.2% | 167 | 28.3% | 0.272 | 0.81 | 0.56 - 1.18 |
| Smoking | 35 | 17.7% | 100 | 16.9% | 0.807 | 1.05 | 0.69 - 1.61 |
| Primipara | 88 | 44.4% | 301 | 50.9% | 0.114 | 0.77 | 0.56 - 1.07 |
| Multipara ≥ 3 | 30 | 15.2% | 65 | 11.0% | 0.120 | 1.45 | 0.91 - 2.30 |
| Maternal BMI < 20 | 34 | 17.2% | 110 | 18.6% | 0.650 | 0.91 | 0.59 - 1.38 |
| Maternal BMI ≥ 35 | 16 | 8.1% | 41 | 6.9% | 0.591 | 1.18 | 0.65 - 2.15 |
| History of cesarean section | 23 | 11.6% | 82 | 13.9% | 0.418 | 0.82 | 0.50 - 1.34 |
| Assisted reproduction therapy | 7 | 3.5% | 25 | 4.2% | 0.668 | 0.83 | 0.35 - 1.95 |
| Maternal hypothyroidism | 2 | 1.0% | 2 | 0.3% | 0.249 | 3.01 | 0.42 - 21.48 |
| Maternal hyperthyroidism | 0 | 0.0% | 1 | 0.2% | 0.562 |  |  |
| Pre-gestational diabetes O24.0, O24.1 | 6 | 3.0% | 9 | 1.5% | 0.179 | 2.02 | 0.71 - 5.75 |
| Gestational diabetes | 12 | 6.1% | 20 | 3.4% | 0.098 | 1.84 | 0.88 - 3.84 |
| Preeclampsia /  high blood pressure | 20 | 10.1% | 55 | 9.3% | 0.741 | 1.09 | 0.64 - 1.88 |
| Oligohydramnios | 6 | 3.0% | 17 | 2.9% | 0.911 | 1.06 | 0.41 - 2.71 |
| Congenital anomalies | 0 | 0.0% | 0 | 0.0% |  |  |  |
| Female sex | 100 | 50.5% | 274 | 46.4% | 0.312 | 1.18 | 0.86 - 1.63 |
| Birthweight < 10% | 10 | 5.1% | 37 | 6.3% | 0.534 | 0.80 | 0.39 - 1.63 |
| Birthweight < 3% | 1 | 0.5% | 10 | 1.7% | 0.218 | 0.29 | 0.04 - 2.32 |
| PPROM | 59 | 29.8% | 138 | 23.4% | 0.070 | 1.39 | 0.97 - 2.00 |
| Induction of labor | 4 | 2.0% | 47 | 8.0% | 0.003 | 0.24 | 0.08 - 0.67 |
| Epidural analgesia | 17 | 8.6% | 74 | 12.5% | 0.134 | 0.66 | 0.38 - 1.14 |
| Emergency cesarean section | 136 | 68.7% | 283 | 47.9% | <0.001 | 2.39 | 1.70 - 3.36 |

BMI, body mass index; PPROM, preterm premature rupture of membranes; NIUT, neonatal intensive care unit
