# Supplementary material for: Impact of fetal presentation on neurodevelopmental outcome in a trial of preterm vaginal delivery: a nationwide, population-based record linkage study
Source: Arch Gynecol Obstet. 2021 Oct 31;306(1):29–35. doi: 10.1007/s00404-021-06146-z (PMC9300511; doi:10.1007/s00404-021-06146-z)
Supplement: Supplementary file 2 — Supplementary file2 (DOCX 15 KB) [file 404_2021_6146_MOESM2_ESM.docx]

**Archives of Gynecology and Obstetrics**

**Impact of fetal presentation on neurodevelopmental outcome in a trial of preterm vaginal delivery: a nationwide, population-based record linkage study**

Toijonen A (University of Helsinki, [anna.toijonen@helsinki.fi](mailto:anna.toijonen@helsinki.fi)), Heinonen S, Gissler M, Seikku L, Macharey G

**Online Resource 2.**

Characteristics of pregnancies that underwent an attempted vaginal delivery in 28+0 to 31+6 gestational weeks 2004-2014 in Finland.

|  | **Breech**  **28+0 - 31+6** | | **Cephalic**  **28+0 - 31+6** | |  |  |  |
| --- | --- | --- | --- | --- | --- | --- | --- |
|  | **n = 401** | **%** | **n = 2460** | **%** | **p-value** | **Odds ratio** | **95% confidence interval** |
| Maternal age < 25 years | 4 | 1.0% | 72 | 2.9% | 0.026 | 0.33 | 0.12 - 0.92 |
| Maternal age ≥ 35 years | 99 | 24.7% | 599 | 24.3% | 0.884 | 1.02 | 0.80 - 1.30 |
| Smoking | 62 | 15.5% | 444 | 18.0% | 0.208 | 0.83 | 0.62 - 1.11 |
| Primipara | 187 | 46.6% | 1278 | 52.0% | 0.048 | 0.81 | 0.65 - 1.00 |
| Multipara ≥ 3 | 52 | 13.0% | 223 | 9.1% | 0.014 | 1.49 | 1.08 - 2.06 |
| Maternal BMI < 20 | 60 | 15.0% | 328 | 13.3% | 0.377 | 1.14 | 0.85 - 1.54 |
| Maternal BMI ≥ 35 | 21 | 5.2% | 128 | 5.2% | 0.978 | 1.01 | 0.63 - 1.62 |
| History of cesarean section | 69 | 17.2% | 344 | 14.0% | 0.089 | 1.28 | 0.96 - 1.70 |
| Assisted reproduction therapy | 14 | 3.5% | 108 | 4.4% | 0.409 | 0.79 | 0.45 - 1.39 |
| Maternal hypothyroidism | 7 | 1.7% | 20 | 0.8% | 0.073 | 2.17 | 0.91 - 5.16 |
| Maternal hyperthyroidism | 1 | 0.2% | 4 | 0.2% | 0.700 | 1.54 | 0.17 - 13.77 |
| Pre-gestational diabetes O24.0, O24.1 | 19 | 4.7% | 78 | 3.2% | 0.108 | 1.52 | 0.91 - 2.54 |
| Gestational diabetes | 26 | 6.5% | 148 | 6.0% | 0.716 | 1.08 | 0.70 - 1.67 |
| Preeclampsia /  high blood pressure | 78 | 19.5% | 388 | 15.8% | 0.064 | 1.29 | 0.98 - 1.69 |
| Oligohydramnios | 3 | 0.7% | 43 | 1.7% | 0.140 | 0.42 | 0.13 - 1.37 |
| Child's female sex | 181 | 45.1% | 1104 | 44.9% | 0.923 | 1.01 | 0.82 - 1.25 |
| Birthweight < 10% | 37 | 9.2% | 192 | 7.8% | 0.331 | 1.20 | 0.83 - 1.74 |
| Birthweight < 3% | 3 | 0.7% | 27 | 1.1% | 0.524 | 0.68 | 0.21 - 2.25 |
| PPROM | 120 | 29.9% | 512 | 20.8% | <0.001 | 1.62 | 1.28 - 2.05 |
| Induction of labor | 9 | 2.2% | 218 | 8.9% | <0.001 | 0.24 | 0.12 - 0.46 |
| Epidural analgesia | 23 | 5.7% | 512 | 20.8% | <0.001 | 0.23 | 0.15 - 0.36 |
| Emergency cesarean section | 291 | 72.6% | 1145 | 46.5% | <0.001 | 3.04 | 2.41 - 3.84 |

BMI, body mass index; PPROM, preterm premature rupture of membranes; NIUT, neonatal intensive care unit
