# Supplementary material for: Impact of fetal presentation on neurodevelopmental outcome in a trial of preterm vaginal delivery: a nationwide, population-based record linkage study
Source: Arch Gynecol Obstet. 2021 Oct 31;306(1):29–35. doi: 10.1007/s00404-021-06146-z (PMC9300511; doi:10.1007/s00404-021-06146-z)
Supplement: Supplementary file 3 — Supplementary file3 (DOCX 15 KB) [file 404_2021_6146_MOESM3_ESM.docx]

**Archives of Gynecology and Obstetrics**

**Impact of fetal presentation on neurodevelopmental outcome in a trial of preterm vaginal delivery: a nationwide, population-based record linkage study**

Toijonen A (University of Helsinki, [anna.toijonen@helsinki.fi](mailto:anna.toijonen@helsinki.fi)), Heinonen S, Gissler M, Seikku L, Macharey G

**Online Resource 3.**

Characteristics of pregnancies that underwent an attempted vaginal delivery in 32+0 to 36+6 gestational weeks 2004-2014 in Finland.

|  | **Breech**  **32+0 - 36+6** | | **Cephalic**  **32+0 - 36+6** | |  |  |  |
| --- | --- | --- | --- | --- | --- | --- | --- |
|  | **n = 1314** | **%** | **n = 18 839** | **%** | **p-value** | **Odds ratio** | **95% confidence interval** |
| Maternal age < 25 years | 25 | 1.9% | 578 | 3.1% | 0.017 | 0.61 | 0.41 - 0.91 |
| Maternal age ≥ 35 years | 304 | 23.1% | 3936 | 20.9% | 0.054 | 1.14 | 1.00 - 1.30 |
| Smoking | 200 | 15.2% | 3290 | 17.5% | 0.038 | 0.85 | 0.73 - 0.99 |
| Primipara | 756 | 57.5% | 9336 | 49.6% | <0.001 | 1.38 | 1.23 - 1.54 |
| Multipara ≥ 3 | 95 | 7.2% | 1907 | 10.1% | <0.001 | 0.69 | 0.56 - 0.86 |
| Maternal BMI < 20 | 135 | 10.3% | 2469 | 13.1% | 0.003 | 0.76 | 0.63 - 0.91 |
| Maternal BMI ≥ 35 | 54 | 4.1% | 831 | 4.4% | 0.606 | 0.93 | 0.70 - 1.23 |
| History of cesarean section | 176 | 13.4% | 2510 | 13.3% | 0.942 | 1.01 | 0.85 - 1.19 |
| Assisted reproduction therapy | 57 | 4.3% | 684 | 3.6% | 0.188 | 1.20 | 0.91 - 1.59 |
| Maternal hypothyroidism | 16 | 1.2% | 169 | 0.9% | 0.239 | 1.36 | 0.81 - 2.28 |
| Maternal hyperthyroidism | 5 | 0.4% | 27 | 0.1% | 0.037 | 2.66 | 1.02 - 6.92 |
| Pre-gestational diabetes O24.0, O24.1 | 47 | 3.6% | 1023 | 5.4% | 0.004 | 0.65 | 0.48 - 0.87 |
| Gestational diabetes | 101 | 7.7% | 1808 | 9.6% | 0.022 | 0.78 | 0.64 - 0.97 |
| Preeclampsia /  high blood pressure | 124 | 9.4% | 1761 | 9.3% | 0.914 | 1.01 | 0.83 - 1.22 |
| Oligohydramnios | 28 | 2.1% | 129 | 0.7% | <0.001 | 3.16 | 2.09 - 4.77 |
| Female sex | 637 | 48.5% | 8161 | 43.3% | <0.001 | 1.23 | 1.10 - 1.38 |
| Birthweight < 10% | 120 | 9.1% | 1361 | 7.2% | 0.010 | 1.29 | 1.06 - 1.57 |
| Birthweight < 3% | 15 | 1.1% | 259 | 1.4% | 0.480 | 0.83 | 0.49 - 1.40 |
| PPROM | 321 | 24.4% | 2916 | 15.5% | <0.001 | 1.77 | 1.55 - 2.01 |
| Induction of labor | 81 | 6.2% | 3967 | 21.1% | <0.001 | 0.25 | 0.20 - 0.31 |
| Epidural analgesia | 199 | 15.1% | 6501 | 34.5% | <0.001 | 0.34 | 0.29 - 0.40 |
| Emergency cesarean section | 695 | 52.9% | 3800 | 20.2% | <0.001 | 4.44 | 3.96 - 4.98 |

BMI, body mass index; PPROM, preterm premature rupture of membranes; NIUT, neonatal intensive care unit
